# Loneliness and cardiovascular disease incidence: two cohorts of older adults in the USA and South Korea

**DOI:** 10.1093/ije/dyaf050

**Published:** 2025-05-08

**Authors:** Harold H Lee, Ruijia Chen, Sakurako S Okuzono, Laura D Kubzansky

**Affiliations:** Department of Biobehavioral Health, The Pennsylvania State University, State College, PA, United States; Department of Epidemiology, Boston University School of Public Health, Boston, MA, United States; Department of Community Health Sciences, UCLA Fielding School of Public Health, Los Angeles, CA, United States; Department of Social & Behavioral Sciences, Harvard T.H. Chan School of Public Health, Boston, MA, United States; Department of Social & Behavioral Sciences, Harvard T.H. Chan School of Public Health, Boston, MA, United States

**Keywords:** loneliness, isolation, cross-national study, cardiovascular disease, health behavior

## Abstract

**Background:**

We investigated the relationship between loneliness and cardiovascular disease (CVD) in older adults from the USA and South Korea. We conducted counterfactual mediation analyses to explore the potential mediation of this relationship by health behaviors.

**Methods:**

We used the Health and Retirement Study (HRS; *n* = 13 073) from the USA and the Korean Longitudinal Study of Aging (KLoSA; *n* = 8311) from South Korea. In both cohorts, baseline loneliness was assessed using one item from the Center for Epidemiologic Studies Depression Scale. Incident CVD was defined as reporting new-onset CVD on the biennial questionnaire or CVD death reported by proxies. Within each cohort, we estimated adjusted hazard ratios (aHRs) of incident CVD according to loneliness (yes/no) over 12–14 years of follow-up, adjusting for baseline covariates: social isolation, sociodemographic factors, health conditions, and health behaviors.

**Results:**

Feeling lonely was associated with an increased likelihood of developing CVD in the USA (aHR: 1.15, 95% CI: 1.04, 1.27) and South Korea (aHR: 1.16, 95% CI: 1.00, 1.34). Several behaviors accounted for a proportion of the association: physical activity (14.3%, *P *=* *0.03 in HRS; 1.3%, *P *=* *0.04 in KLoSA) and alcohol (3.9%, *P *<* *0.001 in HRS; 1.3%, *P *<* *0.001 in KLoSA) in both countries, smoking only in HRS (4.7%, *P *<* *0.001).

**Conclusion:**

The magnitude of the impact of loneliness on CVD was similar in both countries, but behavioral pathways differed. Loneliness may be a risk factor for CVD regardless of culture; however, different prevention strategies in clinical settings may be required.

Key Messages

- The impact of loneliness on cardiovascular disease (CVD) risk seems similar across nations, but the behaviors linking them appear to differ between the USA and South Korea, suggesting cultural factors play a role.
- Loneliness may be a relevant target for CVD prevention strategies in diverse populations, but interventions may need cultural adaptation.
- While the associations are modest, the public health implications of loneliness-related CVD could be significant if a substantial portion of the population experiences loneliness, particularly in the aftermath of the COVID-19 pandemic.

## Introduction

Cardiovascular diseases (CVD) are the leading cause of death worldwide [1]. Extensive epidemiological and biomedical research on CVD has identified behavioral risk factors, including diet, physical activity, tobacco use, and harmful use of alcohol [2–4]. However, these risk factors do not fully account for disease burden and more recent research indicates that various forms of psychological distress, including loneliness, may increase the risk of developing CVD [5, 6].

Loneliness gained recognition as a public health concern with the UK’s Loneliness Minister, amplified by COVID-19 social distancing, and reaffirmed by the US Surgeon General [7, 8]. Loneliness refers to a state of distress in which the actual versus desired social relationships are perceived as insufficient or inadequate. While related to social isolation, loneliness differs in that it arises from a sense of being socially isolated, which can be experienced even by those with objective evidence of social connections (e.g. being married) [9, 10]. A systematic review of empirical studies examining the association between loneliness and CVD published through 2015 [11], identified only four longitudinal studies. Except for one study with a small sample size [12], loneliness was consistently associated with a higher risk of developing CVD, and these associations were generally evident even after controlling for conventional risk factors and numerous potential confounders, including sociodemographics, health conditions, and risk behaviors [13–15]. These findings have been corroborated by several recent well-powered longitudinal studies [16–18], a systematic review in Australia and New Zealand [19], and an extensive systematic review that examined loneliness, social isolation, and mortality risk from 90 cohort studies published between 1982 and 2022 [20]. While the recent systematic review [20] included eight studies not examined in the aforementioned review [11], all studies that examined loneliness and CVD incidence were conducted in Western countries, limiting the generalizability of their findings [21].

The degree to which loneliness increases the risk of developing CVD may vary depending on culture and related social norms. Individuals in East Asian cultures, such as South Koreans, tend to be more interdependent than individuals in Western cultures, such as the USA [22–24]. The intensified social interconnectedness may lead to a more exaggerated psychophysiological response to disturbances in these relationships, as experienced during loneliness, exerting a more pronounced impact on stress-induced CVD. Therefore, the associations between loneliness and CVD incidence may be stronger in countries with high levels of interdependence, such as South Korea.

We examined the association between loneliness and CVD incidence among older adults in the USA and South Korea. We hypothesized that loneliness would be associated with an increased risk of incident CVD in both countries. However, given the stronger interdependent culture in South Korea, we hypothesized that loneliness would be more strongly associated with CVD incidence in South Korea than in the USA. Finally, we conducted formal mediation analyses with health behaviors as putative mediators. Recognizing that the extent to which specific factors serve as mediators may vary across cultural contexts, results from the mediation analyses could provide the scientific basis for developing targeted interventions and policies tailored to the unique social and cultural environments of individuals experiencing loneliness.

## Methods

### Participants

**Figure 1.**
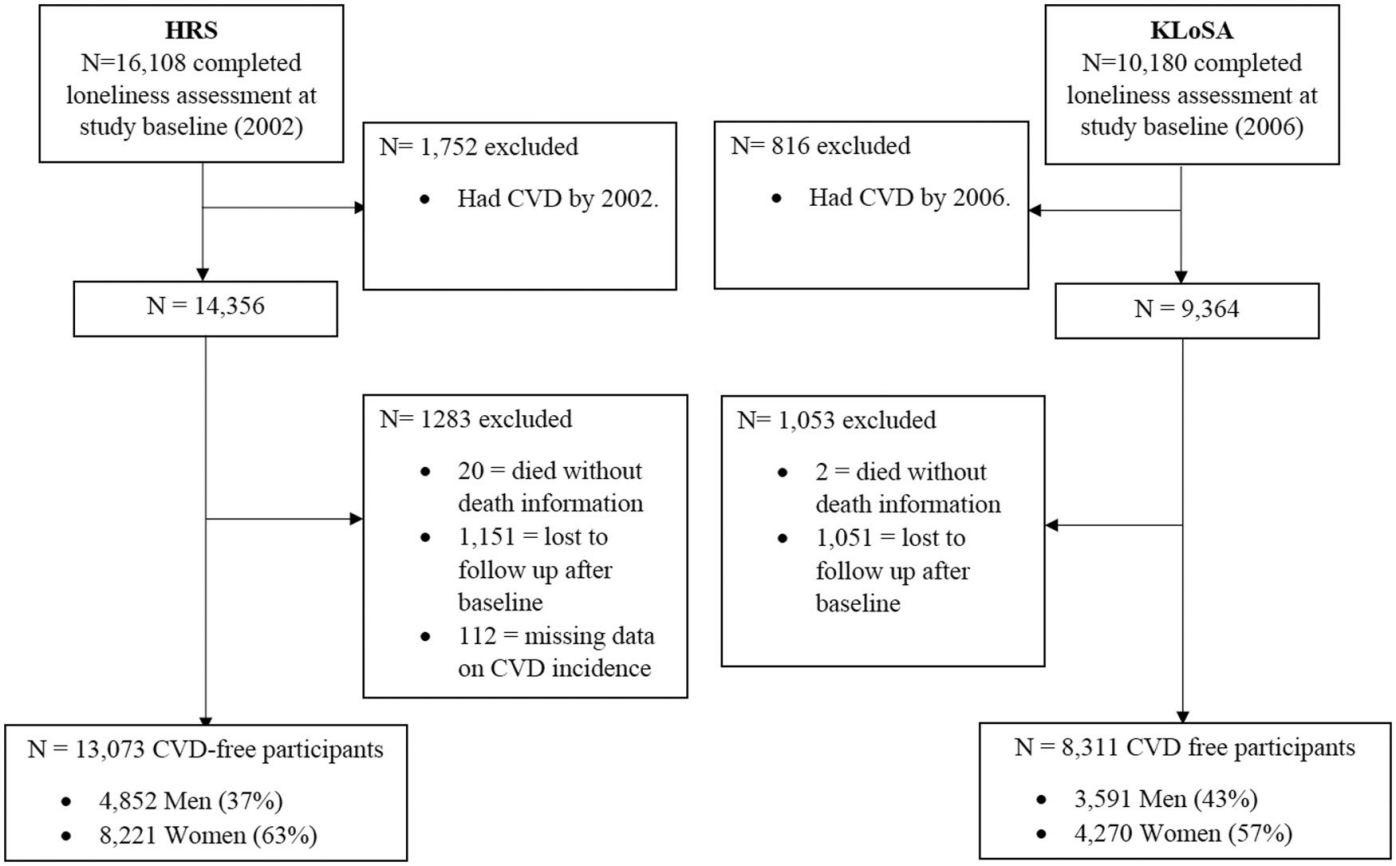
Flow diagram of participant inclusion and exclusion in the Health and Retirement Study (HRS; 2002–2014) and the KLoSA (2006–2018).

Data from the Health and Retirement Study (HRS) and the Korean Longitudinal Study of Aging (KLoSA) were obtained from harmonized data files in the Gateway to Global Aging Data [25]. Both the HRS and KLoSA gather data on aging-related health outcomes at 2-year intervals. Follow-up data including CVD mortality are available in KLoSA through 2018, and in HRS until 2014 the end of data collection included in the Harmonized HRS End of Life Documentation (released in March 2019). To ensure a comparable length of follow-up and general time frame with the KLoSA data, HRS data from waves undertaken during the 2002–2014 period were included, enabling a comprehensive examination of CVD mortality over ∼12 years. Individuals who reported CVD prior to this study’s baseline (2002 in the HRS and 2006 in the KLoSA) and participants missing data on the loneliness question in the Center for Epidemiologic Studies Depression (CES-D) at baseline were excluded. The final analytic sample included 13 073 US adults from the HRS and 8311 South Korean adults from the KLoSA (Fig. 1).

### Measures

#### Loneliness

To facilitate the cross-national comparison, loneliness was operationalized using the same single question about loneliness included in the CES-D scale administered at our study baseline in the HRS (2002) and KLoSA (2006). The single-item measure of loneliness from the CES-D correlates highly with other validated loneliness scales (e.g. the 20-item UCLA loneliness scale) [26] and has been shown to be valid [27, 28]. In HRS, participants responded to the item, “I felt lonely,” with a binary response option (yes/no). In KLoSA, participants responded to the item “I felt lonely” with a 4-point response (0 = none or almost none of the time; 1 = some of the time, 2 = most of the time, and 3 = All or almost all). To facilitate direct comparison with HRS, we recategorized this as a binary variable (0 vs 1–3).

#### Incident CVD

Incident CVD, reports of new-onset CVD or CVD mortality, was assessed from questions asking whether participants were diagnosed with heart attack, coronary heart disease, angina, congestive heart failure, other heart problems, or stroke since the prior follow-up and were included in all biennial questionnaires from 2006 to 2018 in KLoSA and 2002–2014 in HRS. A high correlation between self-reported CVD incidence assessed this way and hospital records is well documented [29, 30], and a previous HRS study confirmed that self-reported stroke is suitable for studying stroke and stroke risk factors [31]. Cause of death data was obtained by proxies identified as close friends or relatives of the deceased and by vital statistics agencies (i.e. National Death Index in the US and National Statistical Office in South Korea).

#### Covariates

Sociodemographic factors include age, gender, race (categorized into Whites, Blacks, and others), ethnicity (Hispanic or non-Hispanic), educational attainment, income, and wealth. Health-related variables included diabetes, hypertension, BMI, smoking status, alcohol consumption, and physical activity. See Supplementary Table S1 for detailed descriptions of sociodemographic factors and health behavioral and health conditions.

Social isolation was assessed using the Berkman-Syme Social Network Index [32] administered in HRS and KLoSA at the study baseline, using information from four domains: (i) marital status, (ii) contact with close friends and relatives, (iii) participation in group activities, and (iv) participation in religious activities. Consistent with prior work [33], we assigned a score of 0 to those who were the least integrated and a score of 3 to those who were the most integrated within each domain, as described in more detail (Supplementary Table S2). Domain scores were summed (range: 0–12) to derive an overall social isolation score used as a continuous variable.

n KLoSA, all covariates had a low missingness (<3%). In HRS, most covariates had low missingness (<3%), except for some subcomponents of the social network index from the Leave-Behind Questionnaire, such as contact with friends and family (18%–23%). For all missing covariates, we conducted multiple imputations by chained equations, a flexible method that also addresses attrition-related issues [34, 35]. Based on the recommendation that the number of imputations should match or exceed the percentage of missing data [36], we performed 30 imputations for the HRS dataset and five imputations for the KLoSA dataset, using the MICE package in R and combined results using the MITOOL package in R [37].

### Statistical analyses

Analyses were conducted separately in KLoSA and HRS and then compared across cohorts. To compute a meta-analytic effect across two cohorts, the estimates were pooled using the *metafor* package in R [38].

To examine the loneliness-CVD incidence relationship, we used Cox proportional hazards models with four increasingly adjusted models (i.e. each model includes covariates from the prior model): (1) age, (2) sociodemographics (gender, income, wealth, education, race/ethnicity in HRS only), (3) social isolation, and (4) health factors (diabetes, hypertension, BMI, smoking, alcohol, physical activity) that may be considered potential confounders or mediators. The Cox models’ proportional hazards assumption for loneliness, which was examined using Schoenfeld residuals [39], was upheld in all models in both countries (*P *>* *0.05).

We conducted mediation analyses considering each health behavior as a potential mediator, adjusting for sociodemographic factors in Model 2. To overcome the limitations of traditional mediation approaches with low statistical power [40, 41], we employed a counterfactual mediation model using the R package *regmedint* [42], which was developed based on SAS macro by Valeri and Vanerweele [43]. This model is flexible and yields higher statistical power by incorporating an exposure-mediator interaction term (e.g. loneliness × smoking) [44].

We conducted several secondary analyses. First, we evaluated potential effect modification by social isolation using a continuous Social Network Index measure; to simplify the interpretation of interactions, we also dichotomized the Social Network Index (i.e. “isolated” vs “not isolated”), using cut points that distinguish ∼15% of the most socially isolated individuals in both countries (i.e. Social Network Index score <4). Second, we evaluated potential effect modification by gender. We tested additive interactions by calculating the relative excess risk due to interaction (RERI) [45] and multiplicative interactions by adding a product term to the Cox proportional hazards model adjusting for sociodemographic factors (i.e. model 2). We also calculated *E*-values [46] to quantify the extent to which an unmeasured confounder would need to be associated with both social integration and mortality to explain away the observed loneliness-CVD risk association. Second, to reduce concerns about reverse causation (i.e. underlying illness could cause changes in loneliness), we conducted an analysis that excluded 1313 HRS participants and 290 KLoSA participants who developed CVD within the first two data collection cycles (i.e. 4 years) of study from baseline (resulting in *n* = 11 891 in HRS and *n* = 8091 in KLoSA); two data collection cycles were chosen to optimize the balance between the need to maintain statistical power while accounting for potential early-stage CVD cases causing loneliness. Third, instead of imputing covariates, we conducted complete case analyses excluding 5402 HRS participants (41%) and 266 KLoSA participants (3%) missing any component of the covariate measures. Since loneliness was not missing at random (Supplementary Table S3), we conducted analyses with datasets in which all covariates, including exposure variable (i.e. loneliness), were imputed (HRS: *n* = 14 296, with 8% missing loneliness data; KLoSA: *n* = 8364 with 0.6% missing in loneliness data). Finally, to ensure the robustness of using baseline weights, we conducted a sensitivity analysis comparing the use of longitudinal versus baseline weights in KLoSA dataset, in which longitudinal weight was available.

All analyses were conducted using R™ (Team, 2016). To account for design effects in each cohort, appropriate survey weights were applied using the *svy* command. To maintain the representativeness of the initial sample, we used the cross-sectional weights available from the baseline for both KLoSA and HRS (longitudinal weights were not available in both cohorts). These weights adjust for selection probability, nonresponse, and poststratification to ensure adequate subgroup representation; cluster IDs account for similarities across people within clusters (i.e. households and geographic areas) to prevent underestimating standard errors [47]. Additive interaction analyses were performed using the *interactionR* package [48], with the delta method for computing the 95% confidence interval [49]. Because survey weights cannot be applied in *regmedint* or *interactionR*, causal mediation and additive interaction analyses were performed without survey weights.

## Results

Compared to South Korean adults, the US adults were older (mean age 66 vs 57 years) and more educated (college graduate: 24% vs 11%). South Korean adults generally had better health behaviors and lower rates of adverse health conditions than the US adults: diabetes (10% vs 14%), hypertension (22% vs 49%), and never smoking (69% vs 43%) at baseline.

At baseline, 16% of the adults in the US sample and 24% of the adults in the South Korean sample felt lonely. During the 12-year follow-up, 25% of HRS and 11% of KLoSA participants developed CVD. The average Social Network Index score was 6.1 ± 2.6 in HRS and 6.3 ± 2.6 in KLoSA. In both countries, those who were lonely also had lower Social Network Index scores, engaged in less physical activity, consumed less alcohol, were more likely to be women, to be less educated, and to have a higher prevalence of diabetes and hypertension (Table 1).

**Table 1.** Sociodemographic characteristics of a nationally representative sample of US adults from the Health and Retirement Study (*n* = 13 073; 2002) and South Korean adults from the KLoSA (*n* = 8311; 2006)^a^

	HRS (USA)		KLoSA (South Korea)	
	Not lonely (*n* = 11 054; 84%)	Lonely (*n* = 2150; 16%)	Not lonely (*n* = 6113; 76%)	Lonely (2198; 24%)
**Demographics**				
Age, mean (SD), years	66.3 (9.1)	68.2 (10.1)	57.2 (9.9)	61.7 (11.3)
Women, %	6720 (58%)	1525 (69%)	3259 (50%)	1461 (63%)
Race				
Whites, %	9241 (89%)	1646 (82%)	n/a	
Black/African American, %	1372 (8%)	392 (12%)	n/a	
Other, %	343 (3%)	110 (5%)	n/a	
Hispanic, %	763 (5%)	274 (11%)	n/a	
Education				
College graduate, %	2471 (29%)	245 (16%)	686 (13%)	94 (5%)
High school graduate, %	6189 (57%)	1092 (56%)	2022 (38%)	387 (22%)
Less than high school, %	2295 (14%)	813 (28%)	3405 (49%)	1717 (72%)
**Health Conditions and Behaviors**				
Diabetes, %	1572 (14%)	406 (18%)	598 (9%)	321 (14%)
Hypertension, %	5377 (48%)	1162 (53%)	1428 (21%)	692 (29%)
Body Mass Index, mean (SD), kg/m^2^	27.4 (5.2)	27.5 (5.9)	23.3 (2.6)	23.1 (3.0)
Smoking, %				
Current smoker	1446 (13%)	399 (19%)	1224 (23%)	410 (21%)
Past smoker	4702 (44%)	888 (38%)	586 (9%)	169 (8%)
Never smoked	4719 (43%)	845 (43%)	4302 (68%)	1619 (72%)
Physical Activity^b^, mean (SD)	0.5 (0.5)	0.4 (0.5)	1.8 (2.8)	1.4 (2.4)
Alcohol consumption^b^, mean (SD), days/week	1.2 (2.1)	0.9 (2.0)	0.8 (1.1)	0.6 (1.0)
Social Network Index^c^, mean (SD)	6.3 (2.6)	4.9 (2.6)	6.6 (2.4)	5.3 (2.7)

a Raw values (i.e. without imputation or survey weights) were used for the values not in parentheses. For % values in parentheses as well as continuous variables (i.e. age and body mass index), survey weights were applied to imputed data.

b Physical activity and alcohol consumption (days/wk) were measured differently in HRS and KLoSA, making cross-national comparisons unreliable. (see Supplementary Table S1 for the description of each measure).

c Berkman–Syme Social Network Index is a composite score calculated using: (i) marital status, (ii) contact with friends/family, (iii) group attendance, and (iv) religious attendance. The score ranges from 0 to 12 with lower score indicating social isolation.

### Associations of loneliness and CVD

Loneliness was associated with a higher risk of developing CVD in both HRS and KLoSA. While the effect size appears to be larger in HRS compared to KLoSA when only adjusting for age (1.31 in HRS vs 1.18 in KLoSA), effect sizes were remarkably similar across models 2 through 4 (Table 2). In the fully adjusted model, feeling lonely was associated with a 16% and 15% higher likelihood of developing CVD in KLoSA and HRS, respectively. The pooled analysis showed no heterogeneity (*Q* = 0.009, *P *=* *0.92). In the meta-analysis of HRS and KLoSA, the fully adjusted HR for loneliness was 1.14 (95% CI: 1.06–1.23).

**Table 2.** Hazard ratios for associations of loneliness (2002 in HRS and 2006 in KLoSA) with major cardiovascular disease incidence (2004–2014 in HRS; 2008–2018 in KLoSA) of a nationally representative sample of US adults from the Health and Retirement Study (*n* = 13 073) and South Korean adults from the KLoSA (*n* = 8311)^a^

			Model 1^b^		Model 2^c^		Model 3^d^		Model 4^e^	
	Person-years	Cases	HR	95% CI	HR	95% CI	HR	95% CI	HR	95% CI
**HRS (USA)**										
Lonely	14 508	554	1.31	1.17, 1.47	1.21	1.09, 1.35	1.18	1.07, 1.31	1.15	1.04, 1.27
Not lonely	84 778	2278	1.00	Ref	1.00	Ref	1.00	Ref	1.00	Ref
**KLoSA (South Korea)**										
Lonely	21 102	365	1.18	1.03, 1.35	1.22	1.06, 1.40	1.19	1.03, 1.39	1.16	1.00, 1.34
Not lonely	60 507	778	1.00	Ref	1.00	Ref	1.00	Ref	1.00	Ref

a Survey weights are applied.

b Adjusted for age.

c Adjusted for Model 1’s covariate and sex, income, wealth, education, race, and ethnicity.

d Adjusted for Model 2’s covariates and social isolation.

e Adjusted for Model 2’s covariates and diabetes, hypertension, body mass index, smoking, alcohol consumption, and physical activity level.

### Role of social isolation in loneliness-CVD associations

No significant multiplicative interaction between loneliness and social isolation was found in HRS (*P *=* *0.11 with categorical social isolation; *P *=* *0.22 with continuous social isolation) or KLoSA (*P *=* *0.43 with categorical social isolation; *P *=* *0.48 with continuous social isolation), nor was any significant additive interaction observed in HRS (RERI=−0.04, 95% CI −0.27 to 0.19) or KLoSA (RERI: 0.04, 95% CI −0.21 to 0.3). Stratified analyses indicate similar trends for socially isolated and nonisolated individuals, though the results are stronger for the nonisolated group (Supplementary Table S4).

### Role of gender in loneliness-CVD associations

No significant multiplicative interaction between loneliness and gender was found in HRS (*P *=* *0.30) or KLoSA (*P *=* *0.56), nor was any significant additive interaction observed in HRS (RERI = 0.08, 95% CI=−0.12 ∼ 0.29) or KLoSA (RERI: −0.15, 95% CI −0.35 ∼ 0.06). Gender-stratified analyses showed consistent estimates between men and women in HRS, with slightly higher estimates for men in KLoSA (Supplementary Table S5).

### Mediation analyses

Table 3 displays the mediation results for all hypothesized mediators, comparing people who did versus did not report feeling lonely. In both samples, physical activity (14.3%, *P *=* *0.03 in HRS; 1.3%, *P *=* *0.04 in KLoSA) and alcohol consumption (3.9%, *P *<* *0.001 in HRS; 1.3%, *P *<* *0.001 in KLoSA) emerged as statistically significant mediators. Notably, an increase in alcohol consumption was associated with lower odds of developing CVD in the full model in HRS (HR = 0.97, 95% CI = 0.94–0.99) and KLoSA (HR = 0.91, 95% CI =0.84–0.98), explaining how fewer days spent on alcohol consumption among lonely individuals had higher odds of developing CVD. Current smoking status was identified as a significant mediator only in the HRS (4.8%, *P *<* *0.001).

**Table 3.** Mediation analyses between loneliness (2002 in HRS and 2006 in KLoSA) and risk of CVD (2004–2014 in HRS; 2008–2018 in KLoSA) of a nationally representative sample of US adults from the Health and Retirement Study (*n* = 13 073) and South Korean adults from the KLoSA (*n* = 8311)^a^

	HRS (USA)		KLoSA (South Korea)	
	Proportion mediated	*P*-value of PNIE	Proportion mediated	*P*-value of PNIE
Smoking	4.8 %	<0.001	2.3 %	0.92
Physical activity	14.3 %	0.03	1.3 %	0.04
Alcohol	3.9 %	<0.001	1.1 %	<0.001

PNIE, pure natural indirect effect expresses how much risk of developing CVD would change on average if the loneliness level was controlled (i.e. fixed) at “not lonely” but the mediator was changed from the level occurring among individuals who reported they were “not lonely” to the level occurring among individuals who reported being “lonely.”

a To ensure temporality between exposure and mediators, we used the three mediators (“smoker,” “physical activity,” “alcohol”) assessed at first wave of follow up after the baseline, which is ∼2 years later.

### Sensitivity analyses

The associations between loneliness and CVD incidence in both countries were moderately robust to unmeasured confounders. For example, the *E*-value in the full model comparing lonely versus nonlonely groups in HRS was 1.44 and 1.59 in KLoSA [50], suggesting that an unmeasured confounder would need to have a minimum HR of 1.44 in HRS and or 1.59 in KLoSA (with both loneliness and CVD risk) to explain away the observed association, above and beyond all of the covariates already considered [46]. The results remained consistent when participants who developed CVD within the first 4 years were excluded (Supplementary Table S6), complete case analyses (Supplementary Table S7), when all covariates, including loneliness, were imputed (Supplementary Table S8), or when longitudinal weights were employed (Supplementary Table S9).

## Discussion

In this cross-national study, longitudinal associations between loneliness and CVD incidence were examined among older adults in two cohort studies, one in the USA and one in South Korea, each with ∼12 years of follow-up. Contrary to the hypothesis that the association would be stronger in South Koreans due to their collectivist culture, feeling lonely was associated with an increased likelihood of developing CVD in both countries, with nearly the same magnitude of 14% higher risk of CVD, controlling for social isolation, sociodemographic factors, health condition, and health behaviors. However, the behaviors linking loneliness and CVD differed somewhat between the USA and South Korea.

The observed effect sizes in both HRS and KLoSA for loneliness in relation to CVD are comparable with those found in prior studies. For example, when evaluating the association of loneliness with incident CVD, a sister study in England, the English Longitudinal Study of Aging, reported a 1.27-adjusted HR over 5 years [18], and a systematic review including studies through 2015 revealed adjusted HRs ranging from 1.31 to 1.53 [13–15] both greater than the adjusted HR observed in the present study. Notably, among studies that accounted for social isolation, a trend toward smaller effect sizes of loneliness emerged (e.g. 1.05–1.06) [16, 17]. While the substantially reduced effect sizes likely result from interconnectedness between loneliness and social isolation, loneliness maintained a modest independent association with cardiovascular health.

The magnitudes of the association of loneliness with incident CVD risk were remarkably similar between South Korean and US adults in our study. Moreover, the links between loneliness and CVD risk were not modified by social isolation or gender. Interestingly, emerging research examining the negative impact of weak social connections on survival in wild mammals shows associations of similar magnitude to what we and others have observed in our study with humans (i.e. odds ratios ranging from 1.23 to 1.72) [51–53], suggesting there may be common mechanisms linking social connection and survival (e.g. hormonal, vascular, immune, and behavioral responses) [54–57], which are evolutionarily conserved. Behavioral pathways by which loneliness affects cardiovascular health differ. For example, smoking was a mediator between loneliness and incident CVD only in the USA, in which smoking has become less socially acceptable in recent decades [58]. In the US context, lonely individuals may experience relatively weaker social pressure or have less motivation to conform to prevailing cultural attitudes related to smoking behavior. These findings highlight the need to consider cultural differences in mechanistic pathways when developing interventions.

While loneliness’s impact on cardiovascular health may be smaller than some conventional CVD risk factors, most cases of disease arise in low-to-moderate risk populations rather than the smaller number of people at high risk (i.e. “prevention paradox”) [59]. Therefore, interventions that can reduce population-level loneliness may result in a significant reduction in CVD incidence in the population. Alongside the population-level approach, targeted interventions for high-risk individuals may be implemented. For example, social prescribing, which connects individuals with community support to enhance their health and well-being [60], offers a viable approach to reducing loneliness within existing public health infrastructures.

Our study has some limitations. We used a single-item measure which has limitations; although important to note is that this item has been validated and correlates with other loneliness scales. Despite the identical content of the loneliness question, the response options differed between HRS and KLoSA, which could limit direct comparisons across findings. This was an observational study. While we took measures to reduce concerns about reverse causation (e.g. removing people with CVD at baseline), unmeasured confounding remains possible. That said, the loneliness-CVD link was still evident even after controlling for a wide array of potential confounders and mediators with a moderately robust *E*-value. The current study also has several notable strengths. First, this is one of the few studies to compare the loneliness-CVD association in Western and non-Western populations using harmonized studies. The harmonization of nationally representative data from two nations provides a unique opportunity for cross-national comparison and generalizable findings.

In conclusion, this study demonstrated that loneliness is associated with an increased risk of developing CVD in both the USA and South Korea. The associations were evident even when different behavioral pathways appeared to be operating, suggesting that loneliness may exert a systemic influence on cardiovascular health independent of behavior. Given that different behavioral pathways seem to operate (i.e. smoking was a mediator only in the USA), we can speculate this may be due to different cultural contexts (e.g. public smoking faces greater stigma in the USA compared to South Korea). Thus, interventions for reducing loneliness may need to be tailored to each specific context for optimal effectiveness. Future research should explore the feasibility of reducing loneliness on a population level and whether such interventions can effectively improve cardiovascular health.

## Ethics approval

Not applicable as HRS and KLoSA datasets are publicly available and deidentified.

## Supplementary Material

dyaf050_Supplementary_Data

## Data Availability

The data underlying this article are available on the Gateway to Global Aging Data website at https://g2aging.org/app/hrd/get-data.
