# Supplementary material for: Loneliness and cardiovascular disease incidence: two cohorts of older adults in the USA and South Korea
Source: Int J Epidemiol. 2025 May 8;54(3):dyaf050. doi: 10.1093/ije/dyaf050 (PMC12060715; doi:10.1093/ije/dyaf050)
Supplement: dyaf050_Supplementary_Data [file dyaf050_supplementary_data.docx]

**Lee et al. Loneliness and Cardiovascular Disease Incidence in Nationally Representative Older Adults in the** **US and South Korea**

Supplements: Table of Contents

[Table S1. Description of Covariates in Health and Retirement Study and Korean Longitudinal Study of Aging 2](#_Toc186101055)

[Table S2 -A . Derivation of Social Integration Score per Berkman-Syme Social Network Index^1^ in Health and Retirement Study 4](#_Toc186101056)

[Table S2- B. Derivation of Social Integration Score per Berkman-Syme Social Network Index^1^ in Korean Longitudinal Study of Aging 5](#_Toc186101057)

[Table S3. Exponentiated Logistic Regression Coefficients (Odds Ratios) and P-values for Missingness in Loneliness Data from the non-imputed raw dataset: Health and Retirement Study (n=14,926) and South Korean adults from the Korean Longitudinal Study of Aging (n=8,364) 7](#_Toc186101058)

[Table S4. Hazard Ratios for Associations of Loneliness with Major Cardiovascular Disease Incidence among Nationally Representative^a^ Samples of older adults from Health and Retirement Study in the U.S. (n=13, 073) and Korean Longitudinal Study of Aging in South Korea (n=8,311) Stratified by Social Isolation. 8](#_Toc186101059)

[Table S5. Hazard Ratios for Associations of Loneliness with Major Cardiovascular Disease Incidence among Nationally Representative^a^ Samples of older adults from Health and Retirement Study in the U.S. (n=13, 073) and Korean Longitudinal Study of Aging in South Korea (n=8,311) Stratified by Sex. 9](#_Toc186101060)

[Table S6. Excluding participants who developed fatal or non-fatal CVD in the first 4 years. Hazard Ratios for Associations of Loneliness with Major Cardiovascular Disease Incidence among Nationally Representative^a^ Samples of older adults from Health and Retirement Study in the U.S. (n=11,760) and Korean Longitudinal Study of Aging in South Korea (n=8,021) 10](#_Toc186101061)

[Table S7. Complete Case Analyses. Hazard Ratios for Associations of Loneliness with Major Cardiovascular Disease Incidence among Nationally Representative^a^ Samples of older adults from Health and Retirement Study in the U.S. (n=7,802) and Korean Longitudinal Study of Aging in South Korea (n=8,045) 11](#_Toc186101062)

[Table S8. Imputed all covariates including loneliness. Hazard Ratios for Associations of Loneliness (2002 in HRS and 2006 in KLoSA) with Major Cardiovascular Disease Incidence (2004-2014 in HRS; 2008-2018 in KLoSA) of a Nationally Representative Sample of U.S. adults from the Health and Retirement Study (n=14,926) and South Korean adults from the Korean Longitudinal Study of Aging (n=8,364) 12](#_Toc186101063)

[Table S9. Comparing the use of baseline vs. longitudinal survey weights in KLoSA. Hazard Ratios for Associations of Loneliness (2006) with Major Cardiovascular Disease Incidence (2008-2018) of a Nationally Representative Sample South Korean adults from the Korean Longitudinal Study of Aging (n=8,311) 13](#_Toc186101064)

# **Table S1. Description of Covariates in Health and Retirement Study and Korean Longitudinal Study of Aging**

Sociodemographic factors include age (year) and sex (male, female). Race, obtained only in HRS, was assessed with the question “What race do you consider yourself to be: White, Black or African American, American Indian, Alaska Native, Asian, Native Hawaiian, Pacific Islander, or something else?” We then recategorized this into 3 categories: Whites, Black/African Americans, and others due to the small sample size in all remaining categories. Ethnicity, also obtained only in HRS, was assessed with the question, “Do you consider yourself Hispanic or Latino?” and coded as Hispanic or non-Hispanic. In HRS and KLoSA, educational attainment was self-reported from 0 to 17 years and then recategorized into 3 categories: college graduate, high school graduate, and less than high school. Income and wealth were assessed in U.S. dollars and Korean won in HRS and KLoSA, respectively, were categorized into quartiles for harmonization. Income in HRS is the sum of the respondent’s wage, salary income, bonuses, overtime pay, commissions, tips, 2^nd^ job or military reserve earnings, and professional practice or trade income. In KLoSA, income includes the after-tax income from wage income and second job earnings. In HRS and KLoSA, total wealth is calculated as the sum of all wealth components minus all debt (KLoSA: total mortgage, total debt, lease safety deposit, safety deposits for other real estate; HRS: total mortgage, home equity line of credit balances and home equity loans, other debts, 2^nd^ home mortgage).

For CVD risk-related health behaviors and health conditions, which can be confounders or mediators in the loneliness-CVD relationship,^33,34^ we included diabetes, hypertension, body mass index (BMI; kg/m^2^ ), smoking, frequency of alcohol consumption, and physical activity, all assessed via self-report on the 2006 questionnaire in each sample. Prior research has validated these health condition measures,^35^ and showed substantial agreement between self-reported health conditions and medical records.^30^ Diabetes and hypertension status were determined in both HRS and KLoSA by asking participants whether a doctor had ever diagnosed them with either condition (yes/no). In HRS and KLoSA, self-reported height and weight were used to derive BMI as a continuous variable (kg/m^2^). Cigarette smoking status was assessed in both HRS and KLoSA: current, former, and latter. The frequency of alcohol consumption was measured by querying the number of days per week drinking alcohol; response options were 0-7 in HRS and 0 (none or < once a month), 1(one to several times a month), 2 (one to several times a week), 3 (most days of the week), and 4 (every day of the week) in KLoSA. In KLoSA, the frequency of exercise participation per week, regardless of intensity, was assessed. In HRS, vigorous physical activity participation (3 or more times a week; yes/no) was assessed.

To satisfy the assumption of temporal ordering in mediation analyses for each health behavior, we used health behavior data obtained 2 years after the baseline. Notably, in 2004, HRS began gathering data on light to moderate-intensity physical activity (3 or more times a week), which was used to create an intensity-weighted sum score (light=1, moderate=2, vigorous=4; range 0-49)^45-47^ for the mediation analysis.

# **Table S2 -A . Derivation of Social Integration Score per Berkman-Syme Social Network Index^1^ in Health and Retirement Study**

|  | 0 | 1 | 2 | 3 |
| --- | --- | --- | --- | --- |
| **Marital status** | “separated”  “divorced”  “separated/divorced” “widowed”  “never married” |  |  | “married”  “married spouse absent” “partnered” |
| **Religious attendance**:  “How often have you attended religious services during the past year? | “not at all” | “one or more times a year” | “two or three times a month” | “more than once a week”, “once a week” |
| **Contact with friends and family:**  Frequency of meeting, speaking, or writing to children, family members, friends. | 4^th^ quartile | 3^rd^ quartile | 2^nd^ quartile | 1^st^ quartile |
| **Group attendance*:**  **2006** “Not including attendance at religious services, how often do you attend meetings or programs of groups, clubs, or organizations that you belong to?” | “never” | “About once a month”  “Less than once a month” | “Once a week”  “2 or 3 times a month” | “More than once a week” |
| **2008a**: “Go to a sport, social, or other club?” | “Not in the last month” | “At least once a month” | “Once a week”  Several times a month” | “Daily”  “Several times a week” |
| **2008b** “Attend meetings of non-religious organizations, such as political, community, or other interest groups?” | “Not in the last month” | “At least once a month” | “Once a week”  “Several times a month” | “Daily”  “Several times a week” |

*Group attendance questions are included in the “Live Behind”, which was asked every other wave to approximately half of the participants in each wave. Accordingly, we used 2008 data when 2006 data was missing. In 2008, group attendance question was asked via two questions (i.e., group attendance 2008a and group attendance 2008b). If participants scored 3 on either 2008a or 2008b, score of 3 was marked. Similarly, if participants scored 2 on either 2008a or 2008b, score of 2 was marked, and so forth.

# **Table S2- B. Derivation of Social Integration Score per Berkman-Syme Social Network Index^1^ in Korean Longitudinal Study of Aging**

|  | 0 | 1 | 2 | 3 |
| --- | --- | --- | --- | --- |
| **Marital status** | “separated”  “divorced”  “widowed”  “never married” |  |  | “married”  “partnered” |
| **Religious attendance**:  Frequency of participating in religious group | “Almost never a year”  “Almost never”  “No participation” | “3 or 4 times a year”  “Once or twice a year” | “”Twice a month”  “Once a month”  “5- or 6 times a year” | “Almost every day”  “2-3 times a week”  “Once a week” |
| **Contact with friends and family:**  Frequency contact with relatives and friends. | “Almost never”  “No close friend or relative” | “twice a month”  “once a month”  “5 or 6 times a year”  “3 or 4 times a year”  “once or twice a year” | “2-3 times a week”  “once a week” | “Almost every day” |
| **Group attendance:**  Added group attendance frequency in each section below:   - social clubs - sports clubs, arts or music groups or classes for senior, - alumni society, society for people from the same hometown, family councils - volunteer groups - political party, NGOs, interest groups - other groups - Everyday = 365 - 1 per week = 1 x 52 = 52 - 2-3 per week = 2.5 x 52 = 130 - 1 per month = 1 x 12 = 12 - 2 per month = 2 x 12 =24 - 1-2 per year = 1.5 x 1 = 1.5 - 3-4 per year = 3.5 x 1 = 3.5 - 5-6 per year = 5.5 x 1 = 5.5 - Almost 0 per year = 0.5 - None = 0 | 4^th^ quartile | 3^rd^ quartile | 2^nd^ quartile | 1^st^ quartile |

**Reference**

1. Berkman LF, Syme SL. Social networks, host resistance, and mortality: a nine-year follow-up study of Alameda County residents. *American journal of Epidemiology*. 1979;109(2):186-204.

# **Table S3.** Exponentiated Logistic Regression Coefficients (Odds Ratios) and P-values for Missingness in Loneliness Data from the non-imputed raw dataset: Health and Retirement Study (n=14,926) and South Korean adults from the Korean Longitudinal Study of Aging (n=8,364)

|  | HRS | | KLoSA | |
| --- | --- | --- | --- | --- |
|  | **Odds Ratio** | **p-value** | **Odds Ratio** | **P-value** |
| Age | 0.98 | **0.00** | 0.99 | 0.62 |
| Sex | 0.19 | **0.00** | 0.72 | 0.41 |
| Race | 0.92 | 0.50 | N/A | N/A |
| Hispanic | 1.22 | 0.30 | N/A | N/A |
| Education | 0.87 | **0.00** | 0.72 | **0.01** |
| Income | 1.00 | 0.85 | 1.00 | 0.09 |
| Wealth | 1.00 | 0.23 | 1.00 | 0.21 |
| Diabetes | 1.18 | 0.25 | 0.82 | 0.70 |
| Hypertension | 1.12 | 0.31 | 0.34 | **0.02** |
| Alcohol | 0.94 | **0.03** | 0.88 | 0.47 |
| Physical activity | 0.87 | 0.22 | 0.92 | 0.26 |
| Body Mass Index | 0.95 | **0.00** | 1.01 | 0.79 |
| Smoking | 0.94 | 0.34 | 0.65 | 0.19 |
| Social Network Index | 1.00 | 0.27 | 0.86 | **0.01** |

Logistic regression models were applied to assess the association between various covariates and the probability of missing data in the binary loneliness variable (coded as 1 for missing data, 0 otherwise). In the Health and Retirement Study (HRS), 8.1% of participants (1,223 out of 14,926) had missing loneliness data, whereas in the Korean Longitudinal Study of Aging (KLoSA), only 0.6% of participants (54 out of 8,364) were missing data for this variable.

# **Table S4.** Hazard Ratios for Associations of Loneliness with Major Cardiovascular Disease Incidence among Nationally Representative^a^ Samples of older adults from Health and Retirement Study in the U.S. (n=13, 073) and Korean Longitudinal Study of Aging in South Korea (n=8,311) Stratified by Social Isolation.

|  | HRS | | KLoSA | |
| --- | --- | --- | --- | --- |
|  | Isolated (n=2,115) | Not isolated (n=10,958) | Isolated (n=1,399) | Not isolated (n=6,912) |
| Cases/PYs | Not Lonely  440 cases/12,024 PYs  Lonely  211 cases/4,604 PYs | Not lonely:  2217 cases, 86,884 PYs  Lonely:  435 cases, 12,323 PYs | Not lonely:  124 cases, 6,612 PYs  Lonely:  118 cases, 5,314 PYs | Not lonely:  654 cases, 53,896 PYs  Lonely:  247 cases, 14,789 PYs |
|  | HR (95% CI) | HR (95% CI) | HR (95% CI) | HR (95% CI) |
| Model 1 ^b^ | 1.10 (0.89-1.37) | 1.34 (1.21-1.49) | 1.06 (0.81-1.39) | 1.18 (0.98-1.44) |
| Model 2 ^c^ | 1.06 (0.85-1.32) | 1.28 (1.15-1.43) | 1.09 (0.83-1.44) | 1.23 (1.01-1.51) |
| Model 3 ^d^ | 1.06 (0.85-1.33) | 1.26 (1.12-1.41) | 1.09 (0.83-1.43) | 1.23 (1.00-1.50) |
| Model 4 ^e^ | 1.02 (0.81-1.28) | 1.23 (1.10-1.38) | 1.05 (0.80-1.38) | 1.18 (0.97-1.44) |

PYs = person-years ^a^ Survey weights are applied

^b^ Adjusted for age

^c^ Adjusted for Model 1’s covariate and sex, income, wealth, education, race, and ethnicity.
^d^ Adjusted for Model 2’s covariates and social isolation.

^e^ Adjusted for Model 2’s covariates and diabetes, hypertension, body mass index, smoking, alcohol consumption, and physical activity level.

# **Table S5.** Hazard Ratios for Associations of Loneliness with Major Cardiovascular Disease Incidence among Nationally Representative^a^ Samples of older adults from Health and Retirement Study in the U.S. (n=13, 073) and Korean Longitudinal Study of Aging in South Korea (n=8,311) Stratified by Sex.

|  | HRS | | KLoSA | |
| --- | --- | --- | --- | --- |
|  | Men (n=4,852) | Women (n=8,221) | Men (n=3,591) | Women (n=4,720) |
| Cases/PYs | Not Lonely  1,229 cases/36,347 PYs  Lonely  206 cases/4,456 PYs | Not lonely:  1,429 cases, 62,560 PYs  Lonely:  442 cases, 12,471 PYs | Not lonely:  403 cases, 27,774 PYs  Lonely:  139 cases, 6,516 PYs | Not lonely:  375 cases, 32,732 PYs  Lonely:  226 cases, 13,585 PYs |
|  | HR (95% CI) | HR (95% CI) | HR (95% CI) | HR (95% CI) |
| Model 1 ^b^ | 1.30 (1.11-1.52) | 1.41 (1.22-1.62) | 1.31 (1.04-1.65) | 1.16 (0.99-1.37) |
| Model 2 ^c^ | 1.20 (1.02-1.41) | 1.22 (1.06-1.41) | 1.43 (1.10-1.85) | 1.32 (1.12-1.56) |
| Model 3 ^d^ | 1.20 (1.02-1.42) | 1.18 (1.02-1.36) | 1.30 (1.01-1.66) | 1.12 (0.94-1.34) |
| Model 4 ^e^ | 1.17 (0.99-1.39) | 1.14 (0.99-1.31) | 1.22 (0.96-1.55) | 1.11 (0.93-1.32) |

PYs = person-years ^a^ Survey weights are applied

^b^ Adjusted for age

^c^ Adjusted for Model 1’s covariate, income, wealth, education, race, and ethnicity.
^d^ Adjusted for Model 2’s covariates and social isolation.

^e^ Adjusted for Model 2’s covariates and diabetes, hypertension, body mass index, smoking, alcohol consumption, and physical activity level.

# **Table S6.** Excluding participants who developed fatal or non-fatal CVD in the first 4 years. Hazard Ratios for Associations of Loneliness with Major Cardiovascular Disease Incidence among Nationally Representative^a^ Samples of older adults from Health and Retirement Study in the U.S. (n=11,760) and Korean Longitudinal Study of Aging in South Korea (n=8,021)

Excluding participants who developed fatal or non-fatal CVD in the first **4 years**

| **4 -years** |  |  | Model 1^b^ | | Model 2^c^ | | Model 3^d^ | | Model 4^e^ | |
| --- | --- | --- | --- | --- | --- | --- | --- | --- | --- | --- |
|  | Person-years | Cases | HR | 95%CI | HR | 95%CI | HR | 95%CI | HR | 95%CI |
| **HRS (United States)** |  |  |  |  |  |  |  |  |  |  |
| Lonely | 16,225 | 347 | 1.26 | 1.11, 1.42 | 1.19 | 1.05, 1.36 | 1.15 | 1.01, 1.32 | 1.12 | 0.98, 1.28 |
| Not lonely | 96,496 | 1645 | 1.00 | Ref | 1.00 | Ref | 1.00 | Ref | 1.00 | Ref |
| **KLoSA (South Korea)** |  |  |  |  |  |  |  |  |  |  |
| Lonely | 21,102 | 365 | 1.25 | 1.08, 1.44 | 1.28 | 1.10, 1.48 | 1.24 | 1.06, 1.45 | 1.21 | 1.04, 1.40 |
| Not lonely | 60,507 | 778 | 1.00 | Ref | 1.00 | Ref | 1.00 | Ref | 1.00 | Ref |

Survey weights are applied

^b^ Adjusted for age

^c^ Adjusted for Model 1’s covariate and sex, income, wealth, education, race, and ethnicity.
^d^ Adjusted for Model 2’s covariates and social isolation.

^e^ Adjusted for Model 2’s covariates and diabetes, hypertension, body mass index, smoking, alcohol consumption, and physical activity level.

# **Table S7.** Complete Case Analyses. Hazard Ratios for Associations of Loneliness with Major Cardiovascular Disease Incidence among Nationally Representative^a^ Samples of older adults from Health and Retirement Study in the U.S. (n=7,802) and Korean Longitudinal Study of Aging in South Korea (n=8,045)

|  |  |  | Model 1^b^ | | Model 2^c^ | | Model 3^d^ | | Model 4^e^ | |
| --- | --- | --- | --- | --- | --- | --- | --- | --- | --- | --- |
|  | Person-years | Cases | HR | 95%CI | HR | 95%CI | HR | 95%CI | HR | 95%CI |
| **HRS (United States)** |  |  |  |  |  |  |  |  |  |  |
| Lonely | 16,225 | 347 | 1.35 | 1.21, 1.51 | 1.29 | 1.14, 1.46 | 1.26 | 1.12, 1.42 | 1.19 | 1.05, 1.36 |
| Not lonely | 96,496 | 1645 | 1.00 | Ref | 1.00 | Ref | 1.00 | Ref | 1.00 | Ref |
| **KLoSA (South Korea)** |  |  |  |  |  |  |  |  |  |  |
| Lonely | 21,102 | 365 | 1.16 | 1.01, 1.35 | 1.21 | 1.05, 1.40 | 1.18 | 1.01, 1.39 | 1.14 | 0.97, 1.33 |
| Not lonely | 60,507 | 778 | 1.00 | Ref | 1.00 | Ref | 1.00 | Ref | 1.00 | Ref |

^a^ Survey weights are applied

^b^ Adjusted for age

^c^ Adjusted for Model 1’s covariate and sex, income, wealth, education, race, and ethnicity.
^d^ Adjusted for Model 2’s covariates and social isolation.

^e^ Adjusted for Model 2’s covariates and diabetes, hypertension, body mass index, smoking, alcohol consumption, and physical activity level.

# **Table S8.** Imputed all covariates including loneliness. Hazard Ratios for Associations of Loneliness (2002 in HRS and 2006 in KLoSA) with Major Cardiovascular Disease Incidence (2004-2014 in HRS; 2008-2018 in KLoSA) of a Nationally Representative Sample of U.S. adults from the Health and Retirement Study (n=14,926) and South Korean adults from the Korean Longitudinal Study of Aging (n=8,364)

|  |  |  | Model 1^b^ | | Model 2^c^ | | Model 3^d^ | | Model 4^e^ | |
| --- | --- | --- | --- | --- | --- | --- | --- | --- | --- | --- |
|  | Person-years | Cases | HR | 95%CI | HR | 95%CI | HR | 95%CI | HR | 95%CI |
| **HRS (United States)** |  |  |  |  |  |  |  |  |  |  |
| Lonely | 18,278 | 711 | 1.29 | 1.17, 1.43 | 1.20 | 1.10, 1.32 | 1.18 | 1.07, 1.30 | 1.14 | 1.04, 1.26 |
| Not lonely | 106,562 | 2933 | 1.00 | Ref | 1.00 | Ref | 1.00 | Ref | 1.00 | Ref |
| **KLoSA (South Korea)** |  |  |  |  |  |  |  |  |  |  |
| Lonely | 20,274 | 370 | 1.17 | 1.02, 1.35 | 1.22 | 1.06, 1.40 | 1.19 | 1.02, 1.39 | 1.15 | 0.99, 1.34 |
| Not lonely | 60,833 | 786 | 1.00 | Ref | 1.00 | Ref | 1.00 | Ref | 1.00 | Ref |

Survey weights are applied

^b^ Adjusted for age

^c^ Adjusted for Model 1’s covariate and sex, income, wealth, education, race, and ethnicity.
^d^ Adjusted for Model 2’s covariates and social isolation.

^e^ Adjusted for Model 2’s covariates and diabetes, hypertension, body mass index, smoking, alcohol consumption, and physical activity level.

# **Table S9.** Comparing the use of baseline vs. longitudinal survey weights in KLoSA. Hazard Ratios for Associations of Loneliness (2006) with Major Cardiovascular Disease Incidence (2008-2018) of a Nationally Representative Sample South Korean adults from the Korean Longitudinal Study of Aging (n=8,311)

|  |  |  | Model 1^b^ | | Model 2^c^ | | Model 3^d^ | | Model 4^e^ | |
| --- | --- | --- | --- | --- | --- | --- | --- | --- | --- | --- |
|  | Person-years | Cases | HR | 95%CI | HR | 95%CI | HR | 95%CI | HR | 95%CI |
| **Baseline weight (r1wtresp)** |  |  |  |  |  |  |  |  |  |  |
| Lonely | 21,102 | 365 | 1.18 | 1.03, 1.35 | 1.22 | 1.06, 1.40 | 1.19 | 1.03, 1.39 | 1.16 | 1.00, 1.34 |
| Not lonely | 60,507 | 778 | 1.00 | Ref | 1.00 | Ref | 1.00 | Ref | 1.00 | Ref |
|  |  |  |  |  |  |  |  |  |  |  |
| **Longitudinal weight (r7lwtresp)** |  |  |  |  |  |  |  |  |  |  |
| Lonely | 20,268 | 370 | 1.17 | 1.03, 1.33 | 1.21 | 1.06, 1.37 | 1.17 | 1.03, 1.34 | 1.13 | 0.99, 1.29 |
| Not lonely | 60,838 | 785 | 1.00 | Ref | 1.00 | Ref | 1.00 | Ref | 1.00 | Ref |

^a^ Survey weights are applied

^b^ Adjusted for age

^c^ Adjusted for Model 1’s covariate and sex, income, wealth, education, race, and ethnicity.
^d^ Adjusted for Model 2’s covariates and social isolation.

^e^ Adjusted for Model 2’s covariates and diabetes, hypertension, body mass index, smoking, alcohol consumption, and physical activity level.
